# *PSCA* polymorphisms and gastric cancer susceptibility in an eastern Chinese population

**DOI:** 10.18632/oncotarget.7137

**Published:** 2016-02-02

**Authors:** Li-Xin Qiu, Lei Cheng, Jing He, Zhi-Rui Zhou, Meng-Yun Wang, Fei Zhou, Wei-Jian Guo, Jin Li, Meng-Hong Sun, Xiao-Yan Zhou, Ya-Nong Wang, Ya-Jun Yang, Jiu-Cun Wang, Li Jin, Xiao-Dong Zhu, Qing-Yi Wei

**Affiliations:** ^1^ Department of Medical Oncology, Fudan University Shanghai Cancer Center, Department of Oncology, Shanghai Medical College, Fudan University, Shanghai, China; ^2^ Cancer Institute, Collaborative Innovation Center for Cancer Medicine, Fudan University Shanghai Cancer Center, Shanghai, China; ^3^ Department of Pediatric Surgery, Guangzhou Women and Children's Medical Center, Guangzhou Medical University, Guangzhou, China; ^4^ Department of Radiation Oncology, Fudan University Shanghai Cancer Center, Department of Oncology, Shanghai Medical College, Fudan University, Shanghai, China; ^5^ Department of Pathology, Fudan University Shanghai Cancer Center, Shanghai, China; ^6^ Department of Gastric Cancer & Soft Tissue Sarcoma Surgery, Fudan University Shanghai Cancer Center, Shanghai, China; ^7^ Ministry of Education Key Laboratory of Contemporary Anthropology and State Key Laboratory of Genetic Engineering, School of Life Sciences, Fudan University, Shanghai, China; ^8^ Fudan-Taizhou Institute of Health Sciences, Taizhou, China; ^9^ Duke Cancer Institute, Duke University Medical Center, and Department of Medicine, Duke University School of Medicine, Durham, NC, USA

**Keywords:** PSCA, polymorphism, gastric cancer, genetic susceptibility

## Abstract

The prostate stem cell antigen (*PSCA*) gene, which encodes a prostate-specific antigen (PSA), was identified as a gene involved in cell adhesion and proliferation. The associations between the *PSCA* rs2294008 and rs2976392 single nucleotide polymorphisms (SNPs) and gastric cancer (GCa) susceptibility were still controversial. To derive a more precise estimation of the associations, we conducted a case-control study of 1,124 cases and 1,192 controls in an eastern Chinese population. We found that the rs2294008T variant genotypes were associated with an increased GCa risk in this study population (CT *vs* CC, OR=1.59, 95% CI=1.33-1.89 and CT+TT *vs* CC, OR=1.38, 95% CI=1.17-1.62). For SNP rs2976392, the variant A genotypes were also associated with an increased GCa risk (AG *vs* GG, OR=1.61, 95% CI=1.35-1.91 and AG+AA *vs* GG, OR=1.47, 95% CI=1.25-1.74). The results were further validated by a meta-analysis. In conclusion, the results indicated that the *PSCA* rs2294008 T and rs2976392 A alleles were low-penetrate risk factors for GCa in this study population. However, large and well-designed studies are warranted to validate our findings.

## INTRODUCTION

Gastric cancer (GCa) is the most frequently occurring cancer and one of the leading causes of cancer-related deaths. There were 951,600 new GCa cases and 723,100 deaths in 2012, accounting for 8% of the cancer cases and 10% of cancer deaths in the world, respectively [1]. Therefore, GCa has become a major public health challenge. While the mechanism of gastric carcinogenesis is still not fully understood, it has been suggested that environmental factors and low-penetrance susceptibility genes may be important in the etiology of GCa. A high rate of *Helicobacter pylori*(*HP*) infection might be a potential risk factor for an increased GCa risk in developing countries (70-90%) than in developed countries (25-50%) [2, 3]. However, only few *HP* carriers will develop GCa; therefore, other factors must play a role in GCa risk. Lifestyle factors such as tobacco smoking, alcohol use and dietary habits are also likely to be potential risk factors for GCa [4]. Although genetic factors for GCa risk are still not fully understood, some recent success in identifying significant associations between genetic variants and GCa risk is encouraging [5-9], and it is necessary to confirm those genetic factors that have been reported to play a role in GCa risk.

The prostate stem cell antigen (*PSCA*) gene, which encodes a prostate-specific antigen (PSA), was identified as a gene involved in cell adhesion, proliferation and patient survival [10, 11]. PSCA is mainly expressed in the region of isthmus/neck, but its expression was undetectable in GCa tumor tissues [12], suggesting a loss of tumor-suppressor effect of PSCA in GCa. In addition, its biological role in cancer advancement was also reported by published *in vivo* functional studies [13, 14]. Therefore, it is necessary to investigate the role of *PSCA* genetic variants in the etiology of GCa. Importantly, several GWAS studies have demonstrated an association between *PSCA* variants and cancer susceptibility [13, 15, 16]. One GWAS study in Korea and Japanese populations reported that two SNPs in the *PSCA* gene (rs2294008 C>T and rs2976392 G>A) were associated with an increased GCa risk [13]. However, these associations were not replicated in the subsequent replication studies [17, 18].

To further confirm the associations between *PSCA* rs2294008 and rs2976392 SNPs and GCa risk, we conducted a replication study in a large eastern Chinese population and also performed a meta-analysis with published studies.

## RESULTS

Baseline characteristics of individuals included in this study were consistent with those described in our previous study [19], but one sample in cases and four samples in controls failed to be genotyped. Thus, the final analysis included 1,124 GCa patients and 1,192 cancer-free controls (Supplemental Table 1). Subjects were well matched by age and sex with more smokers and drinkers in the controls, but these variables were further adjusted in the following multivariate analysis. The rs2294008 and rs2976392 appeared to be in a high linkage disequilibrium (r^2^ = 0.969).

The allele frequencies of SNPs rs2294008 and rs2976392 in cases and controls and their associations with GCa risk are presented in Table 1. The variant rs2294008T genotypes were associated with an increased risk of GCa (CT vs CC, OR=1.59, 95% CI=1.33-1.89 and CT+TT vs CC, OR=1.38, 95% CI=1.17-1.62). For SNP rs2976392, the variant A genotypes were also associated with an increased GCa risk (AG vs GG, OR=1.61, 95% CI=1.35-1.91 and AG+AA vs GG, OR=1.47, 95% CI=1.25-1.74). When these two SNPs were combined, subjects who carried more than one risk alleles exhibited a significantly increased risk of GCa (OR=1.35, 95% CI=1.14-1.59), compared with those who did not carry any risk alleles.

**Table 1:** Logistic Regression Analysis of Associations between *PSCA* Genotypes and Gastric Cancer Risk in an Eastern Chinese Population

Variants	Genotype	Cases(N=1124)	Controls(N=1192)	***P***^a^	Crude OR(95% CI)	**P**	Adjusted OR(95% CI) ^b^	***P*** ^b^
rs2294008								
	CC	537 (47.8)	663 (55.6)	<0.0001^c^	1.00		1.00	
	CT	489 (43.5)	383 (32.1)		1.58 (1.32-1.88)	<0.0001	1.59 (1.33-1.89)	<0.0001
	TT	98 (8.7)	146 (12.3)		0.83 (0.63-1.10)	0.189	0.83 (0.62-1.10)	0.185
	CT/TT	587 (52.2)	529 (44.4)	0.0002^d^	1.37 (1.16-1.61)	0.0002	1.38 (1.17-1.62)	0.0001
rs2976392								
	GG	535 (47.6)	682 (57.2)	<0.0001^c^	1.00		1.00	
	AG	488 (43.4)	388 (32.6)		1.60 (1.35-1.91)	<0.0001	1.61 (1.35-1.91)	<0.0001
	AA	101 (9.0)	122 (10.2)		1.06 (0.79-1.41)	0.713	1.05 (0.79-1.41)	0.723
	AG/AA	589 (52.4)	510 (42.8)	<0.0001^d^	1.47 (1.25-1.73)	<0.0001	1.47 (1.25-1.74)	<0.0001
Combined effect of risk genotypes								
	0	534 (47.5)	654 (54.9)	0.0004^d^	1.00		1.00	
	≥1	590 (52.5)	538 (45.1)		1.34 (1.14-1.58)	0.0004	1.35 (1.14-1.59)	0.0004

CI, confidence interval; OR, odds ratio

a Chi square test for genotype distributions between cases and controls

b Adjusted for age, sex, smoking and drinking status in logistic regression models

c for additive genetic models

d for dominant genetic models

In the stratified analysis presented in Table 2, we found that the associations between the SNP rs2294008 and GCa risk remained significant in dominant models for subgroups of <=59 years (OR=1.53, 95%CI=1.22-1.93), males (OR=1.34, 95%CI=1.10-1.63), females (OR=1.52, 95% CI=1.13-2.06), never smoking (OR=1.54, 95% CI=1.23-1.92), <=25 pack years (OR=1.49, 95% CI=1.06-2.11), never drinking (OR=1.41, 95% CI=1.16-1.70), and NGCA tumor site (OR=1.45, 95% CI=1.21-1.74). We also found that significant associations of rs2976392 with an increased GCa risk remained in the subgroups of age, sex, smoking status, drinking status and tumor site. Consistent with stratified results of rs2294008, the combined effects of these two SNPs on an increased GCa risk were significant, in subgroups of <=59 years (OR=1.49, 95%CI=1.19-1.88), males (OR=1.30, 95%CI=1.07-1.58), females (OR=1.53, 95%CI=1.13-2.07), never smoking (OR=1.48, 95%CI=1.19-1.85), <=25 pack years (OR=1.45, 95% CI=1.02-2.05), never drinking (OR=1.36, 95% CI=1.12-1.65), and NGCA tumor site (OR=1.40, 95% CI=1.17-1.68) (Table 2).

**Table 2:** Stratification analysis for the associations between selected *PSCA* polymorphisms and GC risk

Variables	rs2294008(cases/controls)		Adjusted OR^a^ (95% CI)	*P* ^a^	rs2976392(cases/controls)		Adjusted OR^a^ (95% CI)	*P* ^a^	Combined effect of risk genotypes(cases/controls)		Adjusted OR^a^ (95% CI)	*P* ^a^
	CC	CT/TT			GG	AG/AA			0	≥1	
Median age, yr												
≤59	264/341	314/265	1.53 (1.22-1.93)	0.0003	262/346	316/260	1.61 (1.28-2.02)	<0.0001	262/335	316/271	1.49 (1.19-1.88)	0.0006
>59	273/322	273/264	1.22 (0.96-1.55)	0.098	273/336	273/250	1.34 (1.06-1.70)	0.015	272/319	274/267	1.21 (0.95-1.53)	0.122
Sex												
Males	388/460	412/365	1.34 (1.10-1.63)	0.004	387/476	413/349	1.45 (1.19-1.77)	0.0002	386/452	414/373	1.30 (1.07-1.58)	0.010
Females	149/203	175/164	1.52 (1.13-2.06)	0.007	148/206	176/161	1.59 (1.17-2.15)	0.003	148/202	176/165	1.53 (1.13-2.07)	0.006
Smoking status												
Never	319/346	366/260	1.54 (1.23-1.92)	0.0001	318/350	367/256	1.59 (1.28-1.99)	<0.0001	318/340	367/266	1.48 (1.19-1.85)	0.0005
Ever	218/317	221/269	1.21 (0.94-1.55)	0.134	217/332	222/254	1.35 (1.05-1.74)	0.019	216/314	223/272	1.21 (0.94-1.55)	0.141
Pack-year												
0	319/346	366/260	1.54 (1.23-1.92)	0.0001	318/350	367/256	1.59 (1.28-1.99)	<0.0001	318/340	367/266	1.48 (1.19-1.85)	0.0005
≤ 25 (mean)	104/188	123/165	1.49 (1.06-2.11)	0.023	104/192	123/161	1.59 (1.12-2.25)	0.009	104/185	123/168	1.45 (1.02-2.05)	0.036
> 25 (mean)	114/129	98/104	1.01 (0.68-1.51)	0.948	113/140	99/93	1.23 (0.83-1.83)	0.309	112/129	100/104	1.05 (0.71-1.56)	0.799
Drinking status												
Never	408/475	446/373	1.41 (1.16-1.70)	0.0005	406/485	448/363	1.49 (1.23-1.80)	<0.0001	406/467	448/381	1.36 (1.12-1.65)	0.002
Ever	129/188	141/156	1.32 (0.96-1.82)	0.087	129/197	141/147	1.47 (1.07-2.03)	0.019	128/187	142/157	1.33 (0.96-1.83)	0.084
Tumor site												
GCA	158/663	147/529	1.19 (0. 92-1.53)	0.193	155/682	150/510	1.32 (1.02-1.70)	0.034	155/654	150/538	1.19 (0.92-1.54)	0.176
NGCA	379/663	440/529	1.45 (1.21-1.74)	<0.0001	380/682	439/510	1.53 (1.28-1.83)	<0.0001	379/654	440/538	1.40 (1.17-1.68)	0.0002

a Adjusted for age, sex, smoking and drinking status in logistic regression models

**Figure 1:**
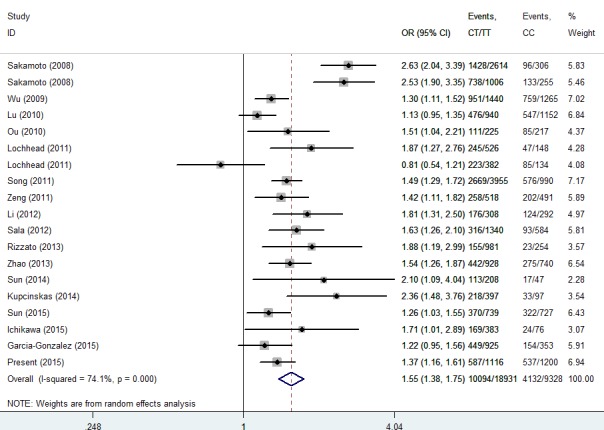
Meta-analysis for the association between *PSCA* rs2294008 SNP and GCa risk in a dominant genetic model

**Figure 2:**
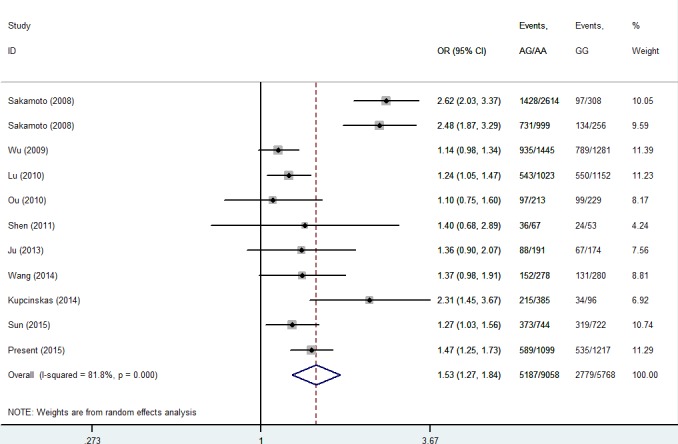
Meta-analysis for the association between *PSCA* rs2976392 SNP and GCa risk in a dominant genetic model

**Figure 3:**
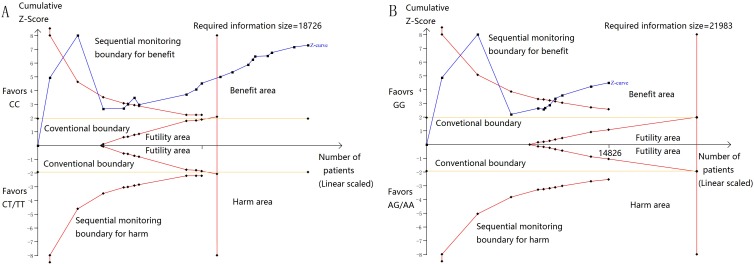
**A.** Sequential meta-analysis for dominant models for rs2294008, with relative risk reduction of 10%, power of 80%, alpha of 5%, and heterogeneity correction of 78.75%. Sequential boundary for benefit has been crossed and the required information size was satisfied. **B.** Sequential meta-analysis for dominant models for rs2294008, with relative risk reduction of 10%, power of 80%, alpha of 5%, and heterogeneity correction of 84.70%. Sequential boundary for benefit has been crossed.

Then, we performed a min meta-analysis, including the present study, of 19 studies [17, 18, 26-42]. Pooled data indicated that both *PSCA* rs2294008 and rs2976392 SNPs were strongly associated with an increased GCa risk (Table 3). For rs2294008 (14226 cases and 14033 controls): heterozygous model: OR=1.52, 95% CI=1.11-2.10; homozygous model: OR=1.75, 95% CI=1.50-2.04; dominant model: OR=1.55, 95% CI=1.38-1.75 (also see Figure 1). For rs2976392 (7966 cases and 6860 controls): heterozygous model: OR=1.48, 95% CI=1.26-1.74; homozygous model: OR=1.60, 95% CI=1.16-2.21; dominant model: OR=1.53, 95% CI=1.27-1.84) (also see Figure 2) without significant publication bias. However, significant heterogeneities across studies were present in these genetic models. Thus, we performed a sensitive analysis to assess the effects of each study on pooled results. Pooled ORs were not affected by omitting each of studies at a time (data not shown), which suggests that the results are robust. The required information size were estimated with 80% power, 5% two-side alpha, 10% reduced relative risk and heterogeneity correlation based on the results of traditional meta-analysis. the TSA analysis suggested that cumulative information size of dominant models for rs2294008 (28259) had reached the cumulative information size (18726). Other meta-analysis did not reach but was not far from the required information size. Moreover, the Lan DeMets sequential monitoring boundary for benefit of wild-type alleles was crossed by cumulative results in all genetic models except for heterozygous models for rs2294008, indicating the results in our meta-analysis are conclusive and reliable. In addition, it was informed by sequential meta-analysis that results of the present study increased the cumulative Z score and therefore strengthened the positive evidence that rs2294008T and rs2976392A variants did have an effect on GCa risk (Figure 3A and 3B).

**Table 3:** Meta-analysis results for the associations between *PSCA* SNPs and GCa risk

Comparison	No. of studies	No. of subjects	OR (95% CI)	*P*	*I*^2^	Model	*P*_bias_
rs2294008							
CT vs. CC	19	22185	1.52 (1.11-2.10)	0.01	95.90%	Random	0.972
TT vs. CC	19	16049	1.75 (1.50-2.04)	<0.001	71.70%	Random	0.506
CT/TT vs. CC	19	28259	1.55 (1.38-1.75)	<0.001	74.10%	Random	0.054
rs2976392							
AG vs. GG	11	12175	1.48 (1.26-1.74)	<0.001	73.40%	Random	0.392
AA vs. GG	11	8419	1.60 (1.16-2.21	<0.001	84.40%	Random	0.392
AA/AG vs. GG	11	14826	1.53 (1.27-1.84)	<0.001	81.80%	Random	0.243

*P*_bias_: *P* value for publication bias

## DISCUSSION

In addition to environmental and lifestyle factors for GCa risk, genetic factors are also important in identifying at-risk populations for prevention of GCa. Although PSCA is mainly expressed in the isthmus/neck region of the stomach where the GCa often occurs [13], it is interesting that PSCA expression is suppressed in GCa tumor tissues. Moreover, it was reported that the expression of PSCA may be coming from some proliferating precursor cells [43]. These findings suggested that PSCA is a potential tumor suppressor in GCa. Therefore, it is biologically plausible that SNPs that lead to down-regulate expression of PSCA make individuals predisposed to GCa. This speculative hypothesis is consistent with or supported by our results that the *PSCA* rs2294008 T and rs2976392 A alleles were associated with an increased GCa risk in the study population. More importantly, these associations were further validated by our meta-analysis with pooled data from all the published studies. Also, results of most pooled data were considered to be robust by sequential meta-analysis, except for rs2294008 with heterogenous results across studies (*I*^2^=95.90%).

However, it was surprising that GCa risk for the subgroups of *PSCA* homozygotes (TT for rs2294008 and AA for rs2297692) were not statistically significant. This may be explained by a co-dominant genetic model, in which only the imbalanced paired protein subunits coded by the two different alleles will have an effect on the proteins’ functions; alternatively, the variant homozygotes may have experienced embryo lethal events, leading to a high rate of abortions; lastly, this finding may be simply due to chance, because of a systemic error in genotyping or because the small sample size of the subgroup may have insufficient statistical power to detect a weak effect or may have generated an unstable risk estimate. All these speculations should be further explored in future larger and mechanistic studies. Therefore, our results should be interpreted with caution.

There are some limitations in the present study. First, although age, sex, smoking and drinking status, and tumor site were taken into consideration for subgroup analysis, other important risk factors such as diet and HP infection, which were missing in this study, might also contribute to the etiology of GCa. Second, new classification of GCa tumor types, which was not available for the patients diagnosed years ago, is also important, which may have a different genetic basis in the etiology. Third, the sample size of the cases in subgroups was largely reduced in the stratification analysis, which may have led to limited statistical power in subsequent analysis.

In summary, our results indicated that the *PSCA* rs2294008 T and rs2976392 A alleles may be low-penetrate risk factors for GCa. However, future studies should incorporate diet, *HP* infection status and Lauren classification to better understand the associations between the *PSCA* SNPs and GCa risk.

## MATERIALS AND METHODS

### Study subjects

This study included GCa patients and cancer-free controls who were part of our ongoing molecular epidemiology study as described previously [19-21]. Briefly, 1,125 unrelated ethnic Han Chinese patients with newly diagnosed and histopathologically confirmed primary GCa were recruited from Fudan University Shanghai Cancer Center (FUSCC) in eastern China between January 2009 and March 2011. Patients other than histopathologically confirmed primary GCa were excluded. In addition, 1,196 age and sex-matched cancer-free ethnic Han Chinese controls were recruited from the Taizhou Longitudinal (TZL) study conducted at the same time period in eastern China as described previously [22]. Blood samples of GCa patients and cancer-free controls were provided by the tissue banks of FUSCC and the TZL study, respectively. All subjects had signed a written informed consent for donating their biological samples to the tissue banks for scientific research. Demographic data and environmental exposure history of each subject were collected. The overall response rate was approximately 91% for cases and 90% for controls. This research protocol was approved by the FUSCC institutional review board.

### SNP genotyping

Using to a standard protocol, we extracted genomic DNA from peripheral blood samples. The rs2294008 and rs2976392 SNPs were genotyped by the TaqMan assay with the ABI7900HT real-time PCR system as reported previously [19]. Subjects’ case-control status was unrevealed in the genotyping process. As recommend by the company, four negative controls (without a DNA template) and two duplicated samples were included in each of 384-plates for the quality control. The assays were repeated for 5% of the samples, and the results were 100% concordant.

### Statistical methods

An individual who never smoked cigarettes was defined as a never smoker; who smoked cigarettes but quit more than one year before diagnosis (for cases) or before the interview (for controls) was defined as a former smoker; and who smoked currently or quit within one year before diagnosis (for cases) or before the interview (for controls) was defined as a current smoker. Ever smokers included both former smokers and current smokers. Those who drank alcoholic beverages at least once a week for one year or more were defined as drinkers, while the others were non-drinkers. The χ^2^ test was used to assess the differences in the distributions of demographic characteristics between cases and controls. The associations between SNPs and GCa risk were assessed by odds ratios (ORs) and 95% confidence intervals (CIs) in heterozygous, homogenous, and dominant models. ORs were calculated by univariate and multivariate logistic regression models. Logistic regression models were used to test for each genetic model with adjustment for age, sex, smoking and drinking status. The combined effect of the tested SNPs on GCa risk was also evaluated in logistic models. Furthermore, associations between the *PSCA* rs2294008 C>T and rs2976392 G>A SNPs and GCa risk were stratified by age, sex, smoking or drinking status, and primary tumor site. All the statistical processes were performed by using SAS software (version 9.1; SAS Institute, Cary, NC)

To further validate our results, we also performed a mini meta-analysis with studies searched from Medline, PubMed and Embase. Principles in search terms and inclusion and exclusion criteria were basically in accordance with previous studies [23, 24]. All primary reports were carefully reviewed, and the relevant references in these papers were also manually searched and reviewed by two independent authors. Then, data were retrieved form included studies and pooled ORs for heterozygous, homozygous, and dominant models were calculated. Heterogeneity among studies was estimated by Chi-square-based Q test. A *P* value greater than 0.10 for the Q-test indicates a lack of heterogeneity among studies, so the pooled OR estimate of the each study was calculated by the fixed-effects model (the Mantel–Haenszel method). Otherwise, the random-effects model (the DerSimonian and Laird method) was used [25]. To validate the stability of the pooled results and find the sources of heterogeneity, we performed the leave-one-out sensitive analysis. Publication bias was shown by the funnel plot, in which the asymmetry was estimated by the Egger's liner regression test, where the statistically significant publication bias was tested out when a *P*<0.05 determined by the *t* test was suggested by Egger. All the statistical processes were achieved by using STATA version 10.0 (Stata Corporation, College Station, TX). Whether results were conclusive could not be answered by traditional meta-analysis without keeping and balancing the type I and type II error. To address this problem, studies included in this meta-analysis were analogous to interim randomized controlled clinical trials. Sequential meta-analysis (SMA) was conducted to calculate the required information size in this meta-analysis. Lan DeMets sequential monitoring boundary was established to control the type I and II error by alpha and beta-spending function methods. Whether the monitoring boundary was crossed was used as a way to estimate the reliability of the results acquired from traditional meta-analysis. All steps were accomplished with the TSA software version 0.9.

## SUPPLEMENTARY MATERIAL TABLE


