# Supplementary material for: PSCA polymorphisms and gastric cancer susceptibility in an eastern Chinese population
Source: Oncotarget. 2016 Feb 2;7(8):9420–8. doi: 10.18632/oncotarget.7137 (PMC4891049; doi:10.18632/oncotarget.7137)
Supplement: Supplementary file 1 [file oncotarget-07-9420-s001.pdf]

PSCA polymorphisms and gastric cancer susceptibility in an Eastern Chinese population

Supplementary Material

Supplemental Table 1. Frequency distribution of demographic characteristics of gastric cancer cases and cancer-free controls.

Variables	Cases No. (%)	Controls No. (%)	P^a
All subjects	1,124 (100.0)	1,192 (100.0)	
Age, yr			0.634
Range	21-86	22-86	
Mean [b]	58.6 ± 11.3	58.7 ± 11.8	
≤ 50	234 (20.8)	267 (22.4)	
51-60	383 (34.1)	384 (32.2)	
61-70	339 (30.2)	372 (31.2)	
>70	168 (14.9)	169 (14.2)	
Sex			0.302
Males	800 (71.2)	825 (69.2)	
Females	324 (28.8)	367 (30.8)	
Smoking status			<0.0001
Never	685 (60.9)	606 (50.8)	
Ever	439 (39.1)	586 (49.2)	
Drinking status			0.008
Yes	270 (24.0)	344 (28.9)	
No	854 (76.0)	848 (71.1)	
Pack-years			<0.0001
0	691 (61.5)	609 (51.1)	
≤ 25 (mean)	220 (19.6)	350 (29.4)	
> 25 (mean)	213 (18.9)	233 (19.5)	
Tumor site			
GCA	305 (27.1)	—	
NGCA	819 (72.9)	—	

GCA, gastric cardia adenocarcinoma; NGCA, non-gastric cardia adenocarcinoma.

[a] Two-sided χ^2 test for distributions between cases and controls.

[b] Data are presented as mean ± SD.
